# Antisense oligonucleotide therapy for patients with Friedreich’s ataxia carrying the c.165+5G>C splicing mutation

**DOI:** 10.1016/j.omtn.2025.102617

**Published:** 2025-07-01

**Authors:** Pouiré Yameogo, Selina Aguilar, Thazha P. Prakash, Frank Rigo, David R. Lynch, Jill S. Napierala, Marek Napierala

**Affiliations:** 1 Department of Neurology, O’Donnell Brain Institute, University of Texas Southwestern Medical Center, 5323 Harry Hines Blvd., Dallas, TX 75390, USA; 2 Ionis Pharmaceuticals Inc., 2855 Gazelle Court, Carlsbad, CA 92010, USA; 3 Department of Pediatrics and Neurology, The Children’s Hospital of Philadelphia, Philadelphia, PA 19104, USA

**Keywords:** MT: Oligonucleotides: Therapies and Applications, Friedreich’s ataxia, antisense oligonucleotides, aberrant splicing, frataxin point mutation, compound heterozygous, mini*FXN* gene

## Abstract

Friedreich’s ataxia (FRDA) is a multisystem, progressive disease. 96% of patients carry biallelic GAA triplet expansion mutations in intron 1 of the frataxin gene (*FXN*). The remaining 4% have a pathogenic GAA expansion on one *FXN* allele and another mutation on the second allele. A point mutation, *FXN* c.165+5G>C, was identified in intron 1 of a patient with FRDA resulting in a significant decrease of FXN levels. Using patient fibroblasts, we demonstrated that the c.165+5G>C mutation affects canonical splicing of *FXN*, leading to the generation of an aberrant transcript. A library of antisense oligonucleotides (ASOs) was designed to target potential intronic splicing regulator motifs and tested in patient cells. Selected O-methoxyethyl (MOE)-ASOs increased FXN levels in c.165+5G>C patient cells without affecting *FXN* splicing in control cells. The leading MOE-ASO increased expression of a mini*FXN* gene carrying the c.165+5G>C point mutation by splicing repair. To increase efficacy, we simultaneously targeted the GAA-expanded allele in patient cells using a synthetic transcription factor (synthetic transcription elongation factor 1 [Syn-TEF1]). This ASO strategy may be therapeutically feasible for patients with FRDA with other point mutations that cause splicing defects. Success in developing treatments for disorders with only a few known cases will give hope to patients with FRDA carrying these rare point mutations.

## Graphical abstract

**Figure:**
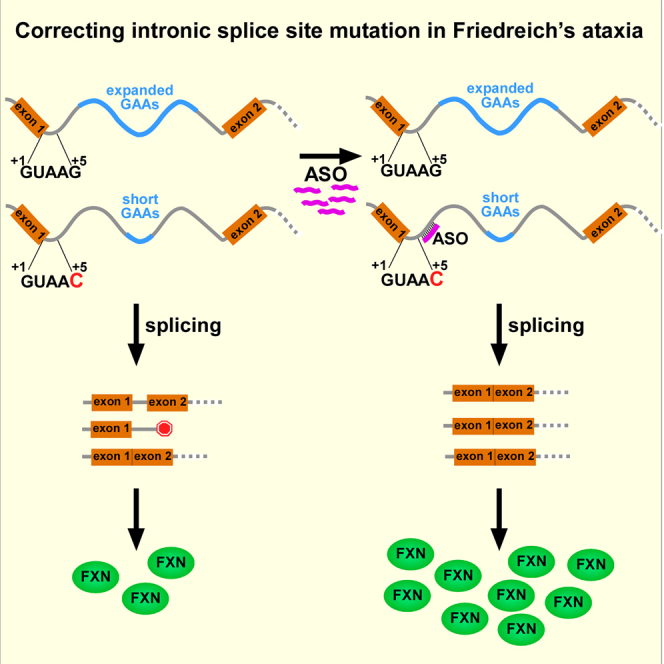

## Teaser

A point mutation in the frataxin (*FXN*) gene (c.165+5G>C) causes a splicing defect that decreases *FXN* expression. Treatment with antisense oligonucleotides (ASOs) covering intronic splicing regulator motifs resulted in increased *FXN* expression from the point mutation allele in Friedreich’s ataxia fibroblasts and from a mini*FXN* gene carrying the same mutation.

## Introduction

Friedreich’s ataxia (FRDA) is a multisystemic, progressive disease, affecting about 1 in 50,000 people worldwide.^1^^,^^2^ The predominant disease-causing mutation is a GAA repeat (GAAr) expansion in intron 1 of the frataxin (*FXN*) gene, located on chromosome 9q13-q21.1.^1^^,^^3^ Unaffected individuals have 7 to 30 GAAr, while patients with FRDA carry 60 to ∼1,700 repeats. Large repeat expansions result in transcriptional silencing of the *FXN* gene and low levels of FXN, a mitochondrial protein involved in iron sulfur cluster biosynthesis and iron metabolism.^4^^,^^5^ Length of the GAAr inversely correlates with FXN levels, with longer repeats resulting in more severe clinical presentation. The expansion mutation is present in the homozygous state in 96% of patients. The remaining 4% of patients with FRDA are compound heterozygous with a pathogenic GAAr expansion on one allele and a non-repeat expansion mutation on the other. More than 60 different *FXN* mutations have been described to date,^6^^,^^7^ including several point mutations predicted to affect sequences important for splicing of the *FXN* pre-mRNA.

Splicing is catalyzed by the spliceosome, a large ribonucleoprotein complex that recognizes splicing signals.^8^ Splice specificity is determined primarily by the sequences of 5′ splice sites (donor), 3′ splice sites (acceptor), the polypyrimidine tract, and the branchpoint. Other *cis*-regulatory elements include exonic and intronic splicing enhancers and silencers.^9^^,^^10^ These splicing regulatory sites are required for efficient exon recognition, particularly when the exon is alternatively spliced or constitutively spliced but has weak splice sites.^11^ Mutations in DNA sequences critical for splicing result in a plethora of defects including reduced splicing efficiency, exon skipping, and intron retention, ultimately producing aberrant transcripts that can be targeted for degradation or translated into aberrant proteins.^12^^,^^13^

Mutations affecting pre-mRNA splicing represent attractive and druggable targets for intervention. One of the best developed approaches to correct these splicing defects is to target the splice region with antisense oligonucleotides (ASOs) to correct aberrant splicing patterns. The action of oligonucleotides requires high complementarity with the target sequence, making ASOs more specific than conventional small-molecule drugs. Various chemical modifications utilized in ASO synthesis further enhance their specificity and increase their stability, allowing for relatively infrequent re-administration of the therapy.^14^ In addition, ASOs have been used in clinical practice for almost 30 years.^15^ Currently, more than 10 synthetic oligonucleotide drugs are approved by the United States Food and Drug Administration (FDA) for treatment of various diseases, and some of them modulate splicing of their target transcripts.^15^^,^^16^ One of the most recognized examples is nusinersen (Spinraza), approved by the FDA for clinical use in 2016 as the first treatment for spinal muscular atrophy and used to treat over 14,000 patients since.^17^ Spinraza targets a splice regulatory element in intron 7 of the survival of motor neuron 2 (*SMN2*) transcript, resulting in incorporation of exon 7 and consequently greater production of the SMN protein. Four different ASOs have been approved by the FDA for treatment of different genetic defects in the Duchenne muscular dystrophy gene via an exon-skipping strategy.^18^^,^^19^^,^^20^ Efforts are also directed toward ASO-mediated individualized treatments by targeting extremely rare or even unique genetic variants.^21^ These *n*-of-1 cases, albeit challenging, offer a real opportunity for individualized ASO treatments for selected, ultra-rare conditions or genetic variants.^22^

As indicated previously, FRDA is a recessive disease, and point mutations, including those potentially affecting splicing, are accompanied by expanded GAA repeats on the second mutated allele.^7^ Importantly, heterozygous mutation carriers are asymptomatic^23^; thus, efficacious correction of point mutation-driven splicing defects would be curative, resulting in an increase of FXN to the carrier level. In addition, the second allele, harboring an expanded GAAr, would still be amenable to any potential repeat-specific therapeutic intervention, such as reactivation of *FXN* expression, with molecules targeting the transcription defect.

Here, we report a combined approach to treat FXN deficiency in a compound heterozygous case of an intronic point mutation and GAAr expansion. Through deep RNA sequencing (RNA-seq), we identified a splicing defect caused by a rare G to C transversion in position 5 of intron 1 (*FXN* c.165+5 G>C). Screening of a library of phosphorothioate (PS), 2′-O-methoxyethyl (2′-MOE)-modified ASOs revealed three ASOs capable of efficacious upregulation of endogenous FXN mRNA and protein in patient-derived fibroblasts. Selected ASOs did not affect canonical splicing of the *FXN* pre-mRNA in control cells. Furthermore, we demonstrated feasibility of concurrent upregulation of *FXN* expression from the allele carrying the expanded GAAr using the synthetic transcription factor, synthetic transcription elongation factor 1 (Syn-TEF1). This ASO strategy may be therapeutically feasible for patients with FRDA with other point mutations that cause splicing defects.

## Results

### *FXN* c.165+5G>C point mutation interferes with canonical splicing of the *FXN* pre-mRNA

**Figure 1:**
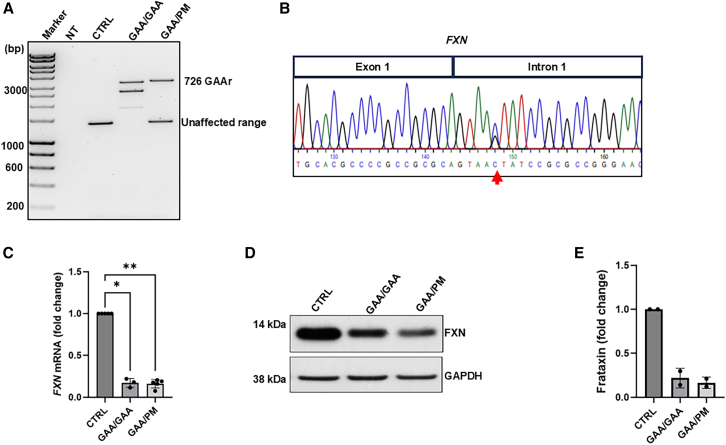
Characterization of FRDA fibroblasts carrying the *FXN* c.165+5G>C point mutation (GAA/PM) (A) Size of the GAA repeats determined by PCR. Analysis of unaffected control (CTRL), GAA/GAA, and GAA/PM fibroblasts. Approximate size of the expanded GAAs is indicated on the right. Multiple bands result from somatic instability of the long repeats. NT indicates no template. (B) DNA sequencing confirmation of the *FXN* c.165+5G>C point mutation (indicated by red arrow). (C) Expression of *FXN* mRNA measured in CTRL, GAA/GAA, and GAA/PM fibroblasts by RTq-PCR. Expression of glyceraldehyde-3-phosphate dehydrogenase (*GAPDH*) mRNA was used as a normalizer. Statistical significance was determined using ordinary one-way ANOVA with post hoc multiple comparisons made to the control mean (Dunnett’s test); significant *p* values are indicated as ∗*p* < 0.05, ∗∗*p* < 0.01. (D) Western blot analysis of frataxin protein expression in CTRL, GAA/GAA, and GAA/PM fibroblasts. GAPDH serves as a loading control. (E) Quantification of western blot data. Data in bar graphs are presented as mean ± standard deviation (SD) (*n* = 2).

Skin fibroblasts from a punch biopsy of a compound heterozygous patient with FRDA carrying the c.165+5G>C point mutation were derived as we described earlier.^24^ We first verified the presence of the point mutation and GAAr expansion allele using PCR. Established fibroblasts (GAA/PM) showed an expanded GAAr of 726 repeats and an allele carrying GAAr within the unaffected range, presumably the point mutation allele (Figure 1A). DNA sequencing of a PCR product spanning an exon 1 and intron 1 fragment confirmed the presence of the *FXN* c.165+5G>C point mutation in one of the two alleles (Figure 1B). The level of *FXN* mRNA and FXN protein in GAA/PM fibroblasts was ∼7-fold lower compared to unaffected control fibroblasts lacking expanded GAAr and comparable with the level of *FXN* mRNA and protein found in FRDA fibroblasts derived from a patient with bi-allelic GAAr expansion (GAA/GAA) (Figures 1C–1E).

**Figure 2:**
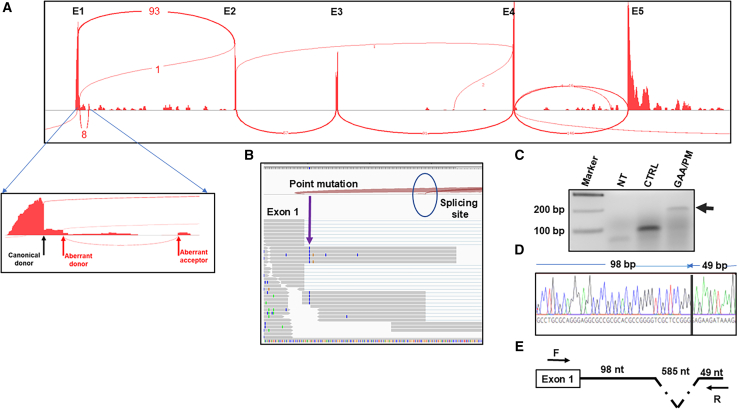
Deep RNA-seq of FRDA *FXN* c.165+5G>C fibroblasts identified an aberrant *FXN* splice isoform in intron 1 (A) Sashimi plot generated by Integrative Genomics Viewer (IGV) illustrating alternatively spliced intron 1 in GAA/PM fibroblasts. A detailed view of the exon 1/intron 1 junction is magnified below. The black arrow indicates the canonical splice donor site, while red arrows designate novel aberrant splice donor and acceptor sites. The novel aberrant donor site is located 98 nt downstream of the exon 1/intron 1 junction and can utilize two different acceptors: (1) 683 nt downstream of the exon 1/intron 1 junction or (2) the canonical exon 2 acceptor. (B) Alignment of RNA-seq reads to the *FXN* sequence. The locations of the point mutation and aberrant splice site are indicated by an arrow and a circle, respectively. (C) Detection of the aberrantly spliced *FXN* transcript in GAA/PM fibroblasts by RT-PCR. The black arrow indicates the aberrant transcript; NT no template control; CTRL, RT-PCR using RNA isolated from unaffected control fibroblasts. (D) Confirmation of the aberrantly spliced transcript by sequencing of the RT-PCR product. (E) Schematic illustration of the aberrant transcript; locations of the primers (F/R) used in the RT-PCR reaction are indicated.

Considering the profound reduction of *FXN* mRNA detected in GAA/PM cells and the location of the point mutation in an intronic region important for pre-mRNA splicing, we conducted deep RNA-seq (>800 M reads). Alignment to the *FXN* gene revealed that all reads spanning the exon 1/intron 1 junction carried the c.165+5 G>C mutation. Furthermore, *FXN* splicing analysis indicated the presence of three different splice variants that included exon 1 and intron 1 sequences: (1) canonical splicing between exon 1 and exon 2, majority of detected products (93) expressed from the second allele lacking the point mutation; (2) novel isoform with partial retention of 98 nucleotides (nt) of intron 1, created by using an aberrant splice donor and the canonical *FXN* splice acceptor site at the intron 1/exon 2 junction (rare, only 1 event detected); and (3) partial retention of 98 nt of intron 1 followed by splicing of 585 nt, created utilizing an aberrant splice donor and aberrant acceptor (Figures 2A and 2B). The latter isoform was detected multiple times (8) in our transcriptome analyses and was predicted to encode a novel, aberrant *FXN* mRNA with a premature termination codon located 49 nt downstream of the aberrant splice acceptor (Figure 2C). We amplified this aberrant mRNA fragment using reverse-transcription PCR (RT-PCR) and confirmed the splicing event using Sanger sequencing (Figures 2D and 2E). In conclusion, we confirmed very low FXN expression in *FXN* c.165+G>C FRDA cells and uncovered an underlying and potentially correctable splicing defect.

### ASOs increase *FXN* mRNA and protein in patient-derived fibroblasts

**Figure 3:**
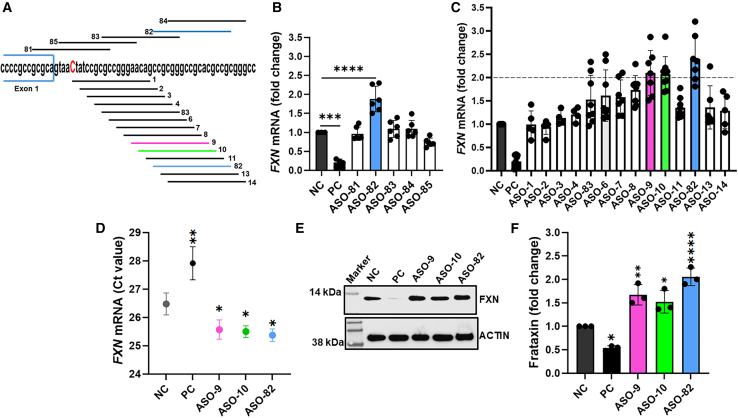
ASOs targeting intron 1 increase frataxin level in *FXN* c.165+5G>C FRDA fibroblasts (A) Locations of the 2′-O-methoxyethyl (2′-MOE) phosphorothioate (PS) ASOs relative to the *FXN* c.165+5G>C point mutation (bold, red). The five ASOs shown above the sequence were used in the initial screen, and ASOs shown below were utilized in the secondary screen. (B) Expression of *FXN* mRNA after transfection with the initial five ASOs. GAA/PM fibroblasts were treated with 30 nM ASOs for 72 h, and *FXN* mRNA levels were measured by RT-qPCR. *GAPDH* expression was used for normalization. A gapmer targeting the *FXN* mRNA was used as a positive control (PC) for transfection, and a non-targeting, scramble 2′-MOE PS ASO was used as a negative control (NC). (C) Expression of *FXN* mRNA after transfection with ASOs from the secondary screen. *GAPDH* expression was used for normalization. A minimum average of 2-fold increase in *FXN* mRNA level was considered as a criterion of success and met by ASOs 9, 10, and 82 (indicated by red, green, and blue bars, respectively). (D) Expression of the *FXN* transcript normalized to total RNA (50 ng) and expressed as raw Ct values as we described in the study by Li et al.^26^ Results for selected ASOs 9, 10, and 82 and controls (NC, PC) are shown. (E) Western blot analysis of frataxin expression in GAA/PM fibroblasts transfected with ASOs 9, 10, 82 and controls (NC, PC). (F) Quantification of frataxin level in GAA/PM fibroblasts transfected with ASOs. Data were normalized to actin and shown relative to NC. Data in bar graphs represent mean ± SD (*n* ≥ 3). Statistical significance for data in (B), (D), and (F) was determined using ordinary one-way ANOVA with post hoc multiple comparisons made to the control mean (Dunnett’s test); significant *p* values are indicated as ∗*p* < 0.05, ∗∗*p* < 0.01, ∗∗∗*p* < 0.001, ∗∗∗∗*p* < 0.0001.

ASOs are a widely recognized therapeutic approach for correction of aberrant splicing.^25^ To identify ASOs able to repair our observed splicing defect, we designed a first set of five ASOs with 2′-MOE ribose and PS backbone modifications targeting the exon 1/intron 1 splicing region (Figure 3A, top; Table S1). The ASOs were transfected to FRDA patient fibroblasts (GAA/PM), and *FXN* expression was determined 72 h later using quantitative RT-PCR (RT-qPCR). Analyses identified that ASO-82 can increase the *FXN* transcript by >1.8-fold (*p* < 0.0001) compared to a negative control (NC) ASO of the same chemical composition (Figure 3B). An ASO gapmer targeting the *FXN* mRNA 3′ UTR was used as a positive control to verify efficient transfection of the ASOs.^26^ Based on the results of the initial screen, we designed a second panel of 12 ASOs in the vicinity of the point mutation (Figure 3A, bottom; Table S1). Transfection followed by RT-qPCR analyses revealed that three ASOs (ASO-9, 10, and again 82) increase *FXN* mRNA ∼2-fold over NC in FRDA GAA/PM fibroblasts (Figure 3C).

As ASO treatment may non-specifically affect expression of RT-qPCR reference genes, we performed additional normalization of the results relative to the total amount of RNA used in the reactions and expressed results as absolute Ct values.^26^ A significant decrease of the Ct values relative to NC was observed for ASOs 9, 10, and 82, confirming the initial RT-qPCR assessment (Figure 3D). Lastly, to determine whether increased *FXN* mRNA translates into FXN protein levels, we performed western blot analyses. An average of 1.5- to 2-fold higher FXN protein levels was detected in GAA/PM fibroblasts treated with ASOs 9, 10, and 82 compared to cells treated with the NC ASO (Figures 3E, 3F, S1A, and S1B), indicating that these ASOs are capable of efficient elevation of FXN in *FXN* c.165+5G>C FRDA cells.

### Efficacy and specificity of ASO-82

**Figure 4:**
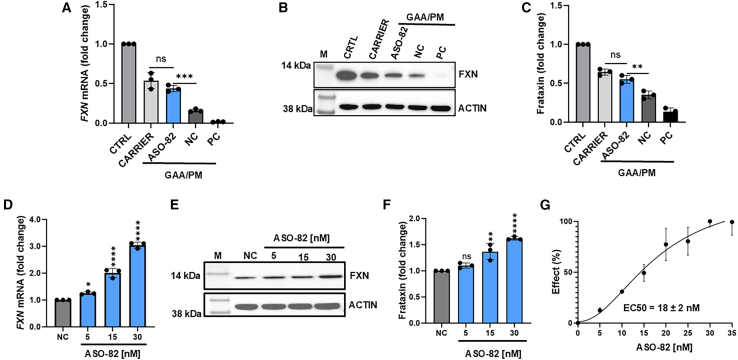
ASO-82 treatment increases frataxin level in *FXN* c.165+5G>C FRDA fibroblasts to carrier levels in a concentration-dependent manner (A) Comparative analysis of *FXN* mRNA expression in GAA/PM fibroblasts upon ASO-82 treatment relative to unaffected control (CTRL; two alleles with GAA tracts <30 repeats) and an asymptomatic expansion carrier (CARRIER; one expanded GAA tract of 711 repeats, one allele with <30 GAA repeats) treated with non-targeting ASO and normalized to *GAPDH* expression. (B) Western blot analysis of frataxin expression in GAA/PM fibroblasts upon ASO-82 treatment relative to CTRL and CARRIER. Actin was used as loading control. M, molecular weight marker. (C) Quantification of frataxin levels relative to CTRL after normalization to actin. (D) The RT-qPCR analysis of *FXN* mRNA expression in GAA/PM fibroblasts 72 h post transfection of ASO-82 at concentrations of 5–30 nM. (E) Western blot analysis of frataxin level in GAA/PM fibroblasts 72 h post transfection of ASO-82 at concentrations of 5–30 nM. Actin was used as a loading control. (F) Quantification of frataxin levels relative to NC after normalization to actin. (G) Dose-response analysis of ASO-82. *FXN* mRNA levels were quantified by RT-qPCR (normalized to *GAPDH*) in GAA/PM fibroblasts transfected with increasing concentrations of ASO-82 in the range of 5–35 nM for calculation of the EC_50_ value. Data in bar graphs represent mean ± SD (*n* = 3). Statistical significance was determined using ordinary one-way ANOVA with post hoc multiple comparisons made to the control mean (Dunnett’s test); significant *p* values are indicated as ∗*p* < 0.05, ∗∗*p* < 0.01, ∗∗∗*p* < 0.001, ∗∗∗∗*p* < 0.0001. For clarity, the statistical significance of selected directly relevant comparisons is presented on the graphs.

Based on repetitive analyses, we selected ASO-82 as the most consistent and efficacious of the ASOs capable of increasing FXN levels when tested using multiple housekeeping genes as normalizers (Figures S2A–S2C). When transfected to GAA/PM fibroblasts, this ASO increased the level of FXN to levels comparable with an asymptomatic carrier (one expanded GAAr allele with 711 repeats and the second allele with GAAr shorter than 30 repeats), reaching approximately 50% of the FXN level observed in an unaffected control (both alleles shorter than 30 GAAr; Figures 4A–4C). We noticed a linear dose response to ASO-82 in *FXN* mRNA and protein in patient cells (Figures 4D–4F). We established the half-maximum effective concentration (EC_50_) of ASO-82 at 18 ± 2 nM based on *FXN* mRNA expression in FRDA c.165+5G>C fibroblasts (Figure 4G).

**Figure 5:**
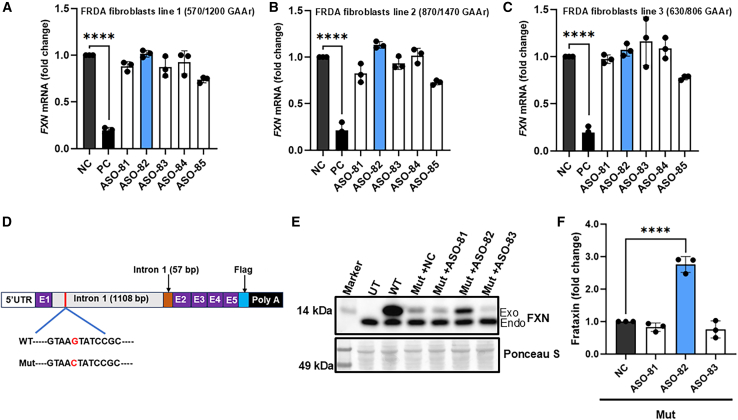
ASO-82-mediated *FXN* mRNA increase is specific to FRDA cells harboring the point mutation (A–C) Three different FRDA fibroblast lines (lines 1–3) with biallelic GAAr expansions were transfected with 30 nM ASOs. The number of repeats in GAA1 and GAA2 alleles is indicated for each line. *FXN* mRNA expression was determined 72 h post treatment and is shown relative to NC after normalization to the expression of *GAPDH* mRNA. (D) Schematic of the mini*FXN* genes used in this study. Purple, exons 1–5; blue, FLAG tag; gray, the first 1,108 bp of *FXN* intron 1; orange, the last 57 bp of *FXN* intron 1, immediately upstream of exon 2. The mini*FXN* gene is expressed using the endogenous *FXN* promoter. Wild type (WT) and mutant (Mut, harboring the *FXN* c.165+5G>C mutation). (E) Western blot analysis of frataxin expression following transfection of WT and Mut mini*FXN* genes to HEK293T cells. The Mut construct was co-transfected with ASOs 81, 82, and 83 or NC, and cells were incubated for 96 h. UT indicates untransfected cells. Ponceau S staining served as a loading control. Endo, signal from endogenous frataxin; Exo, signal from mini*FXN* gene encoding frataxin with a C-terminal FLAG tag. (F) Quantitative analysis of frataxin levels expressed relative to cells transfected with the NC ASO. Ponceau S was used for normalization. Data are expressed as mean ± SD from 3 independent experiments. Statistical significance was determined using ordinary one-way ANOVA with post hoc multiple comparisons made to the control mean (Dunnett’s test); significant *p* values are indicated as ∗∗∗∗*p* < 0.0001.

However, the compound heterozygous status of the patient carrying the point mutation raises the possibility that ASO-82 may increase FXN levels by targeting the expanded GAA allele instead of the point mutation allele. To rule out this possibility, we transfected ASO-82 (selected candidate with the most consistent and robust *FXN* upregulation) into four different FRDA fibroblast lines carrying biallelic GAAr expansions. Analyses performed 72 h after treatment revealed that ASO-82 did not stimulate *FXN* expression in these cells (Figures 5A–5C and S3), confirming specificity of the ASO toward the c.165+5G>C allele. Furthermore, based on the ASO-82 sequence, we designed two additional ASOs carrying mismatches (ASO-82-3 and ASO-82-4, with 3 and 4 mismatches, respectively, Table S1). Transfection of these ASOs into GAA/PM cells did not affect *FXN* mRNA expression, further confirming the specificity of ASO-82 (Figure S2D). Furthermore, RT-PCR analyses revealed that treatment with ASO-82 reduces the aberrant transcript below the level of detection in fibroblasts carrying the *FXN* c.165+5G>C mutation (Figure S4A) without affecting the splicing pattern between exons 1, 2, 3, and 4 of the *FXN* mRNA (Figures S4B and S4C).

We also tested the specificity and efficacy of selected ASOs in mini*FXN* gene constructs harboring wild-type (WT) (mini*FXN*-WT) sequence or the c.165+5G>C point mutation (mini*FXN*-Mut). Each minigene contained a fragment of the human *FXN* promoter, exon 1, truncated intron 1 harboring 1,108 bp of sequence proximal to exon 1 and 57 bp of the sequence proximal to exon 2 (remaining 9,272 bp, including GAA tract, were deleted from the intron), and the remaining coding sequence (exons 2–5) followed by an in-frame FLAG tag and polyadenylation signal (Figure 5D). The mini*FXN* gene was previously demonstrated by us to yield a canonical *FXN* transcript, with correctly spliced truncated intron 1, and to express physiological levels of FXN.^27^ We transfected the WT and Mut mini*FXN* genes into HEK293T cells along with ASOs (ASO-81, ASO-82, ASO-83, and NC) and 96 h later examined mini*FXN* expression by western blot. As expected, expression of mini*FXN*-Mut was significantly lower than WT, but when treated with ASO-82, a significant accumulation of exogenous FXN-FLAG was detected (Figures 5E and 5F). This effect was dose dependent, with 30 nM as the maximum concentration that allowed an increase in *FXN* mRNA without toxicity (Figure S5).

### ASO treatment corrects the splicing defect in a mini*FXN* gene

**Figure 6:**
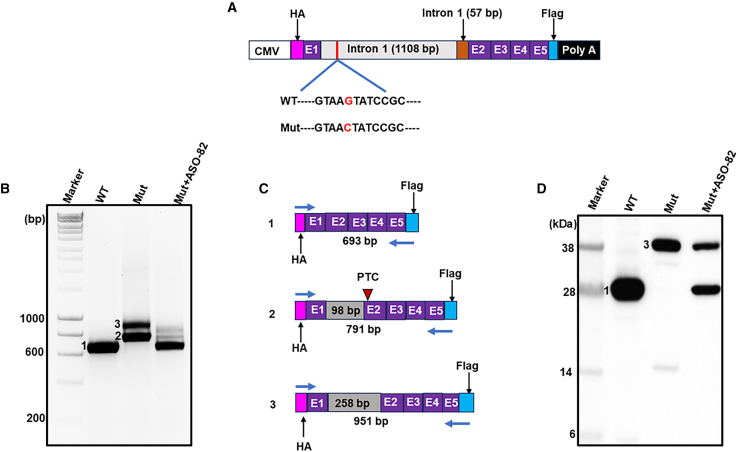
ASO-82 corrects aberrant splicing caused by the *FXN* c.165+5G>C mutation (A) Schematic of a mini*FXN* gene harboring the CMV promoter and an N-terminal HA tag. The remaining *FXN* elements are as described in Figure 5D. (B) HEK293T cells were co-transfected with a mini*FXN* gene (WT or Mut) and 30 nM of ASO-82. RT-PCR analyses were performed using primers located in the sequences encoding the HA (forward) and FLAG (reverse) tags to amplify the entire *FXN* cDNA encoded by the mini*FXN* genes. Three distinct RT-PCR products were amplified and separated by agarose gel electrophoresis: 1 specific for WT mini*FXN*; 2 and 3 specific for Mut mini*FXN*. (C) Schematic representations of DNA sequencing results of RT-PCR products 1, 2, and 3. The length of each product and the length of retained intron 1 in products 2 and 3 are indicated. PTC, premature termination codon identified in product 2. Locations of HA and FLAG tags and RT-PCR primers are indicated. (D) Western blot analysis of frataxin expression after transfection with mini*FXN* gene and ASO-82. The blot was probed with an anti-HA antibody to visualize the N-terminal fragment of each protein. Note the size difference between endogenous, mature FXN (∼14 kDa) and mini*FXN*-encoded FXN (∼28 kDa).

Detection of the splicing defect and its ASO-mediated correction is challenging in an endogenous setting due to low expression of the *FXN* transcript produced from the c.165+5G>C point mutation allele, in the presence of the more abundant canonical transcript produced from the second allele harboring expanded GAAs. In fact, our deep RNA-seq data revealed 10 times more reads mapped to the canonical *FXN* mRNA than to the aberrantly spliced transcript produced from the c.165+5G>C allele (93 versus 9 reads, Figure 2A). To verify that ASO-82 treatment indeed corrects the splicing defect in a cellular system lacking the overpowering signal produced from the second, expanded GAA allele, we utilized constructs expressing a modified mini*FXN* gene. To increase expression of the mini*FXN* gene, we replaced the endogenous *FXN* promoter with a strong CMV promoter. In addition to the C-terminal FLAG tag, we introduced an N-terminal HA tag to enable the detection of N-terminal FXN peptides potentially produced from the aberrantly spliced transcripts. Mitochondrial import is associated with double cleavage of the FXN precursor to the mature form that results in removal of the N-terminal 80 amino acids. Therefore, monitoring the effect of the c.165+5G>C mutation on the FXN protein is enabled by specific detection of the N terminus using an epitope tag. Two CMV promoter-driven mini*FXN* genes (WT and Mut, Figure 6A) were introduced to HEK293T cells followed by RNA and protein analyses 72 and 96 h post transfection, respectively. The WT mini*FXN* expressed a single mRNA of ∼693 nt encoding for canonical *FXN* mRNA with correctly spliced intron 1 (Figure S6). On the other hand, introduction of the c.165+5G>C point mutation in the Mut construct resulted in synthesis of two transcripts, none of them harboring the canonical *FXN* mRNA sequence (Figures 6B and 6C). One of the isoforms (∼791 nt long; Figure 6B, product 2) retained 98 nt of intron 1 sequence (exactly the same sequence that was retained in FRDA c.165+5G>C fibroblasts; Figure S7), while the second, ∼951 nt long (Figure 6B, product 3), retained 258 nt of intron 1 (Figure S8). Next, we determined which proteins are encoded by the mini*FXN* constructs by western blot analysis. WT mini*FXN* expressed the expected full-length FXN precursor (∼26 kDa); however, Mut mini*FXN*, despite two distinct transcripts, expressed a high level of only one, significantly larger protein (Figure 6D). This protein is likely a product of translation of transcript 3 (Figures 6B and 6C), as transcript 2 is predicted to encode a premature stop codon resulting in the production of an unstable, truncated protein. Importantly, co-transfection of the Mut mini*FXN* with ASO-82 corrected the aberrant splicing at the RNA level (more than 95% of the correct RNA, Figure 6B; lane Mut+ASO-82) and protein levels (more than 65% of correct protein; Figure 6D; lane Mut+ASO-82), demonstrating high efficacy and specificity of the treatment.

### Simultaneous targeting of both point mutation and expanded GAAr alleles restores FXN expression to the level of unaffected control cells

**Figure 7:**
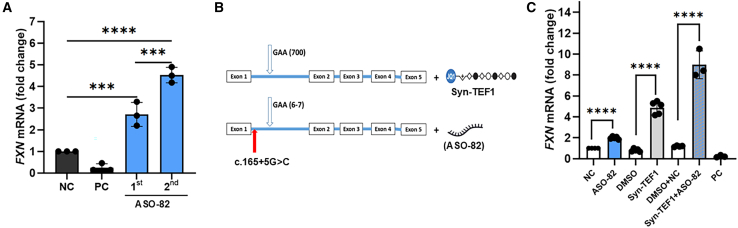
Simultaneous targeting of both point mutation and expanded GAAr alleles significantly upregulated frataxin expression (A) RT-qPCR analysis of *FXN* expression following repeated transfections of GAA/PM-PM fibroblasts with 30 nM of ASO-82. RNA was extracted from all samples 72 h after the first transfection. 1^st^, *FXN* mRNA expression measured after one transfection; 2^nd^, *FXN* mRNA expression measured after two transfections within a 48 h interval. *FXN* expression was normalized to *GAPDH* and expressed relative to NC. Results are expressed as mean ± SD from 3 independent experiments. (B) Schematic depicting simultaneous targeting of GAA/PM cells with point-mutation-specific ASO-82 and the GAA expansion-specific transcription inducer Syn-TEF1.^29^ (C) Relative expression of *FXN* mRNA, normalized to *GAPDH* mRNA, in GAA/PM cells treated with 30 nM of ASO-82 (control NC), 1 μΜ of Syn-TEF1 (control DMSO), and 30 nM of ASO-82 + 1 μΜ of Syn-TEF1 (control NC and DMSO). The *FXN*-specific gapmer (PC) was used to determine the efficiency of transfection. Results are expressed as mean ± SD (*n* ≥ 3). Statistical significance in (A) was determined using ordinary one-way ANOVA with post hoc multiple comparisons made to the control mean (Dunnett’s test), while statistical significance in (C) was determined using unpaired, two-tailed t tests between each treatment and its appropriate control; significant *p* values are indicated as ∗∗∗*p* < 0.001, ∗∗∗∗*p* < 0.0001.

One of the most challenging limitations of ASO therapy is achieving an appropriate intracellular concentration without inducing toxicity. To test whether continuous delivery of ASO-82 would increase FXN levels, we performed repetitive transfections of this ASO. A 30 nM dose of ASO-82 was used in both treatments separated by 48 h. Total RNA was isolated 16 h after the second transfection, and *FXN* mRNA level was determined using RT-qPCR. A significant boost of *FXN* transcript, from ∼2.5-fold to almost 5-fold over control (non-targeting ASO also delivered two times), was detected, demonstrating that increased availability of ASO-82 can further upregulate *FXN* expression (Figure 7A). Thus, exploring strategies that increase ASO bioavailability could improve the efficacy of the treatment.^28^

Although treatment with ASO-82 resulted in a significant *FXN* increase compared to the NC, even up to carrier levels, it alone did not achieve the level measured in unaffected control cells (both GAA tracts below 30 repeats; Figures 4A–4C). The patient with FRDA carrying the c.165+5G>C point mutation is a compound heterozygote, with a second allele bearing a GAAr expansion. Thus, independent, parallel targeting of the GAAr expansion using a repeat-specific approach should allow for a more robust upregulation of *FXN* expression. To test this, we treated c.165+5G>C fibroblasts with ASO-82 along with Syn-TEF1 (Figure 7B). Syn-TEF1 is a modified GAAr-specific polyamide capable of specific binding to expanded GAA repeats (polyamide domain) and recruitment of transcription elongation machinery via a covalently linked JQ1 molecule.^29^ Targeting both alleles with different agents resulted in a significant, almost ∼10-fold increase in *FXN* mRNA levels over baseline, reflecting a considerable cumulative effect (Figure 7C). This demonstrates that synergistic strategies can be used in compound heterozygote patients to elicit the maximal increase of endogenous *FXN* expression in patient cells.

## Discussion

RNA splicing is an integral component of gene expression in eukaryotic cells. Precise splicing permits translation of correct proteins, and alternative splicing increases diversity of transcriptomes and proteomes, while aberrant splicing may result in development of diseases. Correct removal of the intronic sequence depends on protein splicing machinery and appropriate regulatory RNA sequences, as well as a conducive chromatin environment.^9^^,^^30^ Mutations in regulatory regions, especially in the vicinity of splice donors and acceptors, may have detrimental consequences.

Described herein, a c.165+5G>C transversion in the human *FXN* gene is an example of an intronic single-nucleotide substitution evoking aberrant splicing of the *FXN* transcript, leading to FRDA. The fifth nucleotide of the intron is a part of the 5′ splice recognition sequence, and in most transcripts, this position is occupied by a G and pairs with C at the 5′ end of the U1 small nuclear RNA during splicing. Thus, typically, mutations of this residue are associated with loss of donor recognition, aberrant splicing, and pathological consequences.^31^^,^^32^ Indeed, in the case of the *FXN* c.165+5G>C mutation, we found profoundly decreased levels of the mutated transcript, indicating activation of nonsense-mediated decay. Retention of 98 nt of intron 1, as identified by deep RNA-seq, results in a premature stop codon 722 nt downstream of the point mutation. The pathogenic effect in this patient with FRDA is likely caused by the overall decrease of FXN, from both the point mutation and expanded GAAr alleles. However, we cannot entirely rule out that the short transcript carrying the point mutation leads to the accumulation of a toxic protein product. In addition, different transcripts resulting from aberrant splicing could be produced in a tissue-specific manner. Experiments with mini*FXN* genes identified a transcript with aberrant retention of 258 nt of intron 1 (in frame with the remaining *FXN* coding sequence), resulting in the translation of a mutated, significantly larger FXN. While we did not detect any aberrant proteins in FRDA *FXN* c.165+5G>C fibroblasts, the antibodies used in our studies recognize the C-terminal portion of FXN and thus would not detect such N-terminally truncated proteins. An antibody-free mass spectrometry approach will be necessary to assess aberrant protein production from the *FXN* c.165+5G>C allele in patient tissues.^33^

A survey of Genome Aggregation Database revealed 42 different sequence variants within the initial 10 bp of introns 1–4 and the last 10 bp of introns 1–4 (in proximity to putative splicing regulatory regions) of the *FXN* gene. While the clinical significance of these variants is unknown, our study demonstrates that one of them has a clear pathological impact. In the case of autosomal recessive diseases, clinical presentation of a variant can only be assessed in the presence of the second mutated allele (i.e., GAAr expansion in FRDA). However, considering the relatively common presence of heterozygous individuals carrying expanded GAAs on one of the alleles (1:50–100 in the Caucasian population), future reports of novel compound heterozygotes associated with splicing defects can be expected. For example, there have been three individuals identified carrying the *FXN* c.165+5G>C mutation, as well as another individual carrying a *FXN* c.165+1G>A mutation that affects the same splice donor sequence.^7^

More than 2,000 diseases with autosomal recessive inheritance have been described so far. A survey of 508 genes associated with 450 autosomal recessive diseases revealed ∼47,000 putatively pathogenic sequence variants, with almost 10% of them being splice variants. On one hand, these variants represent a very large pool for potential therapeutic intervention; on the other hand, individually these variants are very rare, with allele frequencies below 0.001%. In the future, some recessive conditions may benefit from a “universal” treatment, such as gene therapy or protein supplementation, while some will require individualized treatments that target a specific mutation.

Here, using a two-step screening strategy, we identified ASO-82, targeting the intronic sequence in the vicinity of the point mutation, capable of upregulating FXN to the levels compared to asymptomatic heterozygote carriers (approximately 50% of FXN found in unaffected, non-carrier cells). Moreover, we demonstrated that the ASO reestablishes the canonical splicing pattern characteristic for WT *FXN* transcript without affecting the GAAr expansion allele. Mechanistically, it is likely that ASO-82 blocks a potent intronic splicing silencer sequence via a mechanism similar to that described for treatment of an intronic mutation of inhibitor of kappa light polypeptide gene enhancer in B cells, kinase complex-associated protein, located 6 nt downstream of exon 20 of this gene.^34^ A similar approach was also utilized to correct aberrant splicing caused by the c.2657+5G>A mutation in intron 16 of the cystic fibrosis transmembrane conductance regulator gene.^35^ ASO technology has seen great progress in recent years and is currently in clinical practice for treating various diseases.^14^ Furthermore, several FDA and European Medicines Agency-approved oligonucleotide therapies target splicing. In this work, we utilized chemically modified ASOs (PS-MOE), identical to the Spinraza (nusinersen) splice-switching ASO approved for treatment of spinal muscular atrophy.^36^ These chemical modifications improve ASO stability, target affinity, and bioavailability and enhance cellular uptake, distribution, and half-life.^14^^,^^21^^,^^37^ A limitation of this study is the use of lipid-mediated methods for ASO delivery. Follow-up studies will be conducted in FRDA patient-derived induced pluripotent stem cell-differentiated post-mitotic neurons utilizing gymnotic uptake to deliver ASOs. While the therapeutic potential of an ASO/oligonucleotide candidate can typically be rigorously and robustly demonstrated through *in vitro* testing, efficient *in vivo* delivery and cellular uptake often hinder the full therapeutic impact of oligonucleotide drugs. For further development of ASO-82 as a therapeutic agent, *in vivo* delivery strategies targeting the central nervous system (CNS), particularly the eyes, would be most advantageous for patients with FRDA carrying the c.165+5G>C mutation. Methods of local delivery, such as direct injection of oligonucleotides into the cerebrospinal fluid or intravitreal injection, are effective in distributing ASOs throughout the CNS or delivering therapeutic doses of the ASO to the target organ.^38^^,^^39^ Bioconjugation strategies can also increase the potential for specific sub-targeting within an organ or tissue by enhancing ASO stability or its interaction with specific receptors.^21^^,^^40^ Packaging in nanoparticulate carriers, such as lipid nanoparticles or exosomes, can facilitate the uptake of ASOs to specific tissues, including brain, when coupled with a targeted delivery method.^41^^,^^42^^,^^43^ Finally, cell-penetrating peptides can be used to enhance oligonucleotide uptake across multiple tissues as a direct conjugate or via nanoparticle packaging, which is an important therapeutic consideration for a systemic disease like FRDA.^44^^,^^45^^,^^46^

FRDA heterozygous patients represent a small subset (∼4%) of all FRDA cases. Moreover, we can estimate that only approximately 12% of the point mutations resulting in clinical presentation cause splicing defects, making the target population even smaller.^7^ In most cases, the same point mutation can be found in a few patients with FRDA, but sometimes only a single affected person is identified. However, as shown in the example of milasen, an ASO designed to treat a specific defect in a patient with Batten disease, multidisciplinary efforts can result in developing a therapy for a single patient.^47^ Developing *N* = 1 ASOs for treatment of unique defects is challenging, and recent guidelines presented by the *N* = 1 collaborative initiative help facilitate this process, as more individualized ASO-based therapies will certainly be pursued in the future.^48^

With one exception,^49^ all patients with FRDA diagnosed thus far carry at least one allele with expanded GAA repeats. Hence, targeting a point mutation allele can be accompanied by a repeat expansion-specific therapy. We demonstrated that stimulation of *FXN* transcription using the GAA-specific Syn-TEF1 polyamide leads to a significant increase of *FXN* expression, achieving levels observed in control individuals. Applying ASO therapy specific for a point mutation does not preclude targeting the expanded allele or addressing FXN deficiency via gene therapy or protein supplementation. While the later approaches are currently undergoing clinical trials,^50^ the path for approvals of ASOs has been explored successfully in the past and may represent a straightforward, complementary approach. Patients carrying point mutations may benefit from combination therapy approaches that simultaneously target the point mutation-specific defect and general FXN insufficiency by gene therapy, protein delivery, or targeting expanded GAAs.

## Materials and methods

### Cell lines

HEK293T cells were cultured in Dulbecco’s modified Eagle’s medium (DMEM) (Life Technologies, Cat# 11965092) supplemented with 10% fetal bovine serum (FBS; GE Healthcare Life Sciences, Cat# SH30910.03) at 37°C, 5% CO_2_. For primary fibroblast cell line derivation, skin biopsy samples were obtained, and all procedures were conducted in accordance with approvals of Children's Hospital of Philadelphia (IRB no.: 10-007864) and UT Southwestern Medical Center (IRB STU-2023-0478). Human primary fibroblasts were cultured in DMEM (Life Technologies, Cat# 11965092) supplemented with 15% FBS and 1% non-essential amino acids (Thermo Fisher Scientific, Cat# 11140050), as we described earlier.^24^^,^^26^

### Oligonucleotide synthesis

2′-MOE, PS, and 5-methylcytosine ASOs were synthesized by Integrated DNA Technologies, Inc., and dissolved in nuclease-free water (Invitrogen, Cat# AM9937) to a stock concentration of 100 μM. A list of oligonucleotide sequences is provided in Table S1.

### Plasmid construction

An *FXN* c.165+5G>C point mutation was introduced using site-directed mutagenesis into pLenti-FXNL-HA-hygR harboring the miniF*XN* gene mini*FXN*_7.^27^ Subsequently, derivatives of mini*FXN*_7 WT and c.165+5G>C mutant were cloned into pCR4 and pcDNA 3.1/Hygro (+) (Invitrogen, Cat# V870-20) as follows.

#### Plasmid pCR4_miniFXN_7-165+5G>C construction

The pcR4_mini*FXN*_7^27^ was digested using ClaI and AgeI, and the backbone fragment of 6,392 bp (F1) was purified using a QIAquick Gel Extraction Kit (QIAGEN, Cat# 28706). A PCR fragment (F2) encoding the point mutation was obtained using pLenti-FXNL-HA-hygR_165+5G>C as a template with primers:

FW-Mut 5′ CGTGGCCTGCGCACCGACATCGATGCGACC.

RW-Mut 5′ CAGCCGCACACCCCTCGGAACCGGTCCCCT.

NEBuilder HiFi DNA Assembly Master Mix (New England Biolabs, Cat# E2621S) was used to assemble F1 and F2.

#### Plasmids HA_miniFXN_7 and HA-miniFXN_7-165+5G>C construction

A 6,461 bp fragment obtained after Bsu36I and ClaI digestion of pcR4_mini*FXN*_7 and pcR4_miniFXN_7-165+5G>C (termed F-A-WT and F-A-Mut, respectively) was purified using a QIAquick Gel Extraction Kit (aforementioned). A PCR fragment containing a small part of exon 1 (F-B) was obtained using pCR4_mini*FXN*_7 as a template with the primers:

FW-HA 5′ CTCCTGGCGTCACCCAGCCCAG.

RW-HA 5′ GGGGCGTGCAGGTCGCATCGATG.

A gBlock of 210 bp containing the HA epitope sequence (F-C) was obtained from IDT, Inc. NEBuilder HiFi DNA Assembly was used to assemble fragments F-A-WT + F-B + F-C (HA_mini*FXN*_7) and F-A-Mut + F-B + F-C (HA-mini*FXN*_7-165+5G>C).

#### Plasmids CMV-HA_miniFXN_7 and CMV-HA_miniFXN_7-165+5G>C construction

These plasmids were constructed using three DNA fragments. A PCR fragment (Fα) was amplified using plasmid pCR4_mini*FXN*_7 as a template and primers:

FW-PolyA 5′ GAATTTAGCGGCCGCGACGTCG.

RW-PolyA 5′ AATTCACTAGAGTCGCAGATCC.

A PCR fragment (Fβ) of the pcDNA 3.1/Hygro (+) vector was amplified using primers:

FW-CMV 5′ GGATCTGCGACTCTAGTGAATTGACATTGATTATTGACTAG.

RW-CMV 5′ CCCGCTCCGCCCTCCAGCCAGTAAGCAGTGGGTTCTCTAG.

The third fragment was generated by AatII and AfeI digestion of the HA_mini*FXN*_7 (F-γ fragment) and HA-mini*FXN*_7-165+5G>C (F-γ-Mut fragment) and agarose purification of the 6,071 bp band.

NEBuilder HiFi DNA Assembly was used to assemble fragments Fα, Fβ, and F-γ (CMV-HA_mini*FXN*_7) and F-γ/F-γ-Mut (CMV-HA_mini*FXN*_7-165+5G>C).

The DNA sequence of all constructs was verified by Nanopore sequencing (https://www.plasmidsaurus.com/home) and analyzed with SnapGene 7.1.2 software. For transfection experiments, plasmids were isolated using the Plasmid Plus Midi Kit (QIAGEN, Cat# 12943).

### Plasmid and ASO transfection

HEK293T cells were transfected with Lipofectamine 2000 (Thermo Fisher, Cat# 11668019) according to the manufacturer’s recommendations. A day before transfection, cells were seeded in DMEM (100,000 cells/per one well of a 24-well plate, 500,000 cells per one well of a 6-well plate). Transfection was performed using 500–2,500 ng of plasmids and 5–40 nM of ASOs. ASOs were always delivered to cultured cells using Lipofectamine 2000-mediated transfection. Cell culture medium was replaced 24 h after transfection. Cells were collected 72 h–96 h after transfection.

Lipofectamine RNAiMAX (Fisher Scientific, Cat# 13-778-150) was used to transfect primary fibroblast lines with ASOs. Fibroblasts were seeded into 6-well plates at a density of 160,000 cells per well the day before transfection. Nine microliters of Lipofectamine RNAiMAX and 5–35 nM of ASO were prepared in 300 μL of OptiMEM medium (Thermo Fisher Scientific, Cat# 31985070) per each well. The medium was replaced 24 h later, and cells were analyzed 48 h–96 h after transfection. For Syn-TEF1 treatment, commercially synthesized Syn-TEF1 (Pharmaron, Inc.; purity >95% by HRMS and HPLC) was dissolved in dimethyl sulfoxide (DMSO) (Sigma, Cat# D8418), diluted in sterile water, and added directly to the culture medium to achieve a final concentration of 1 μΜ.

### RT-PCR

For mRNA analysis, total RNA was extracted from HEK293T cells and treated with DNase I (New England Biolabs, Cat# M0303), then cDNA was synthesized using SuperScript VILO cDNA Synthesis Kit (Thermo Fisher Scientific, Cat#: 11754050). Superscript VILO RT enzyme and buffer were added to a total volume of 20 μL, and reverse transcription was carried out for 10 min at 25°C, followed by 60 min at 42°C and 5 min at 85°C. cDNA fragments were amplified by PCR. PCR was performed with CloneAmp HiFi PCR Premix (Takara Bio Inc., Cat# 639298) using primers specified for the mini*FXN* gene listed in Table S2. The PCR conditions were as follows: an initial denaturation of 30 s at 98°C, 34 cycles of 10 s at 98°C, 15 s at 60°C, and 30 s at 72°C follow by 5 min at 72°C of final elongation. PCR products were analyzed on 1.5% agarose gels and documented using a ChemiDoc MP Imaging System (Bio-Rad).

### Quantitative real-time RT-PCR

Total RNA was extracted using the RNeasy Mini Kit (QIAGEN, Cat# 74134) and treated with DNase I (TURBO DNA-free; Thermo Fisher Scientific, Cat# AM1907) according to the manufacturer’s protocol. The RT-qPCR reactions were performed using the iTaq Universal SYBR Green One-Step Kit (Bio-Rad, Cat# 172–5151) and Bio-Rad CFX96 real-time PCR system. Reverse transcription was conducted at 50°C for 10 min, followed by initial denaturation for 1 min at 95°C. This was followed by 40 cycles of denaturation at 95°C for 10 s and annealing at 60°C for 20 s. All reactions were performed in triplicate. Control reactions were also performed without reverse transcriptase to confirm removal of genomic DNA. All primers used for RT-qPCR analyses are listed in Table S2.

### Sanger sequencing

The PCR products for sequencing were purified using a PCR Purification Kit (QIAGEN, Cat# 28104) and submitted for Sanger sequencing (Eurofins Genomics, LLC). Alignment of the sequences was performed using SnapGene 7.1.2 software and Benchling (https://benchling.com/).

### Western blot

Proteins were extracted using a buffer containing 0.1% NP-40 (Igepal), 0.25 M sodium chloride, 5 mM EDTA, and 50 mM of 4-(2-hydroxyethyl) piperazine-1-ethanesulfonic (pH 7.5) supplemented with 1% protease inhibitor cocktail (Sigma-Aldrich, Cat# P8340) at the time of extraction. Protein concentration was determined by Bradford Protein Assay Kit (Bio-Rad, Cat# 5000006). Thirty to fifty micrograms of whole-cell extract were electrophoresed on NuPAGE 4%–12% Bis–Tris gels (Fisher scientific, Cat# NP0321BOX) or NuPAGE 12% Bis Tris Protein Gel (Fisher Scientific, Cat# NP0341BOX) followed by transfer onto nitrocellulose membranes (Bio-Rad, Cat# 165–0112). After transfer, membranes were stained with Ponceau S (Sigma-Aldrich, Cat# P3504) and imaged using a ChemiDoc MP Imaging System (Bio-Rad). The following primary antibodies were used: anti-FXN (Proteintech Group, Cat# 14147-1-AP) at 1:1,000; anti-ACTIN (Santa Cruz, Cat# SC-47778) at 1:2,000; anti-GAPDH (Millipore, Cat# MAB374) at 1:10,000; and anti-HA (Cell Signaling Technology, Cat# 3724) at 1:1,000. Secondary antibodies used are as follows: anti-rabbit (GE Healthcare Life Sciences, Cat# NA934V) and anti-mouse (GE Healthcare Life Sciences, Cat# NA931V) at 1:5,000. Signals were quantified using Image Lab 6.1 software (Bio-Rad). The SeeBlue Plus2 Pre-stained Protein Standard was used to estimate molecular weights (Invitrogen, Cat# LC5925).

### GAAr PCR

Amplification of GAAr expansions in the *FXN* gene was performed by PCR using GAAr expansion primers (Table S2) with the FailSafe PCR System (Epicentre, Cat# FS99250; mix D Epicentre, Cat# FS99250) and 100 ng of genomic DNA. Reaction conditions were described earlier.^51^ The PCR products were analyzed on 1% agarose gels, and GAAr size was determined as described in the study by Long et al.^52^

### RNA-seq

RNA was isolated from fibroblasts using the RNeasy Plus Mini Kit (QIAGEN, #74134). Genomic DNA was removed using the TURBO DNA-free Kit (above). Sequencing libraries were generated at Novogene Co., Ltd., using NEBNext Ultra TM RNA Library Prep Kit for Illumina (NEB, USA) following manufacturer’s recommendations. A total of 843 M high-quality reads were generated in the deep RNA-seq experiment. High-quality reads were aligned to the genome GRCh38 using HISAT2. FeatureCounts (v.1.5.0-p3) was used to count read numbers mapped to each gene. rMATS 4.1.0 was used to analyze alternative splicing events. The Integrative Genomics Viewer application (IGV_2.16.2) was used for visualization and generation of sashimi plots at the *FXN* locus.^53^ RNA-seq data are available from GEO (accessionGEO: GSE262763).

### Statistical analysis

All experiments were performed multiple times, with *n* ≥ 3. Statistical analyses were performed using GraphPad Prism 10.3.0. The number of replicates and *p* values are included in the figure captions accompanying the data. FXN expression in a given cell line or upon treatment follows normal distribution as determined by Kolmogorov-Smirnov and Shapiro-Wilk tests. Therefore, unless otherwise indicated, to determine statistical significance, ordinary one-way ANOVA tests were performed with post hoc multiple comparisons made to the indicated control mean using the Dunnett’s test correction.

## Data availability

Further information and requests for resources, data, and reagents should be directed to Marek Napierala (marek.napierala@utsouthwestern.edu). All unique/stable reagents generated in this study and datasets are available with a completed materials transfer agreement. RNA-seq data are available from GEO (accession GEO: GSE262763).

## Acknowledgments

This study was supported by the 10.13039/100000090Congressionally Directed Medical Research Programs (CDMRP) under award no. HT9425-23-1-0337 (to M.N.); 10.13039/100000002National Institutes of Health, 10.13039/100000065National Institute of Neurological Disorders and Stroke R01NS081366 and R01NS121038 (to M.N.); and 10.13039/100002108Friedreich's Ataxia Research Alliance (to M.N. and independently to J.S.N.). Research in the Napierala Laboratory is also supported by the Dear High Risk-High Reward Research Fund in Neurodegenerative Diseases from Margaret Dear.

## Author contributions

P.Y. and S.A. performed the majority of experiments and analyzed the data. J.S.N. derived, established, and characterized the fibroblast lines. D.R.L. provided patient samples and consulted on the project. T.P.P. and F.R. provided ASOs. P.Y., S.A., J.S.N., and M.N. designed the experiments. P.Y., J.S.N., and M.N. wrote the paper. J.S.N. and M.N. acquired funding. All authors read the article, contributed comments and suggestions, and approved the final version.

## Declaration of interests

T.P.P. and F.R. are employees of Ionis Pharmaceuticals, Inc. The company had no influence on the performed studies, data analysis, and experimental conclusions.

## Supplemental information

Document S1. Figures S1–S8 and Tables S1 and S2

Document S2. Article plus supplemental information
