# Supplementary material for: Antisense oligonucleotide therapy for patients with Friedreich’s ataxia carrying the c.165+5G>C splicing mutation
Source: Mol Ther Nucleic Acids. 2025 Jul 1;36(3):102617. doi: 10.1016/j.omtn.2025.102617 (PMC12284527; doi:10.1016/j.omtn.2025.102617)
Supplement: Document S1. Figures S1–S8 and Tables S1 and S2 [file mmc1.pdf]

OMTN, Volume *36*

Supplemental information

Antisense oligonucleotide therapy

for patients with Friedreich's ataxia carrying

the c.165+5G>C splicing mutation

Pouiré Yameogo, Selina Aguilar, Thazha P. Prakash, Frank Rigo, David R. Lynch, Jill S. Napierala, and Marek Napierala

Supplemental Figures

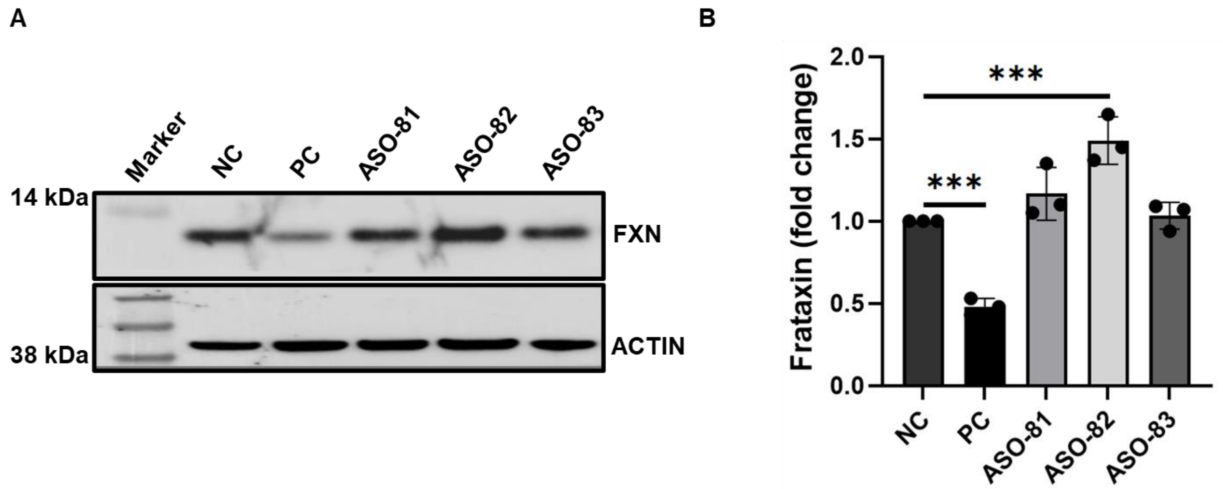

Figure S1: ASO-82 increases frataxin level in FRDA c.165+5G>C fibroblasts. A) Western blot analysis of frataxin expression in GAA/PM fibroblasts transfected with 30 nM ASOs. Actin is used as a loading control. **B**) Quantification of frataxin protein level in GAA/PM fibroblasts transfected with ASOs. Data were normalized to Actin. Image Lab software (Bio-Rad) was used to quantify frataxin signal relative to the negative control (NC) ASO. Data in bar graphs represents mean values ± standard deviation (SD) (n ≥ 3. Statistical significance was determined using one-way ANOVA with post-hoc multiple comparisons to the control mean (Dunnett's test), with significant *p* values indicated as: ***$p<0.001$.

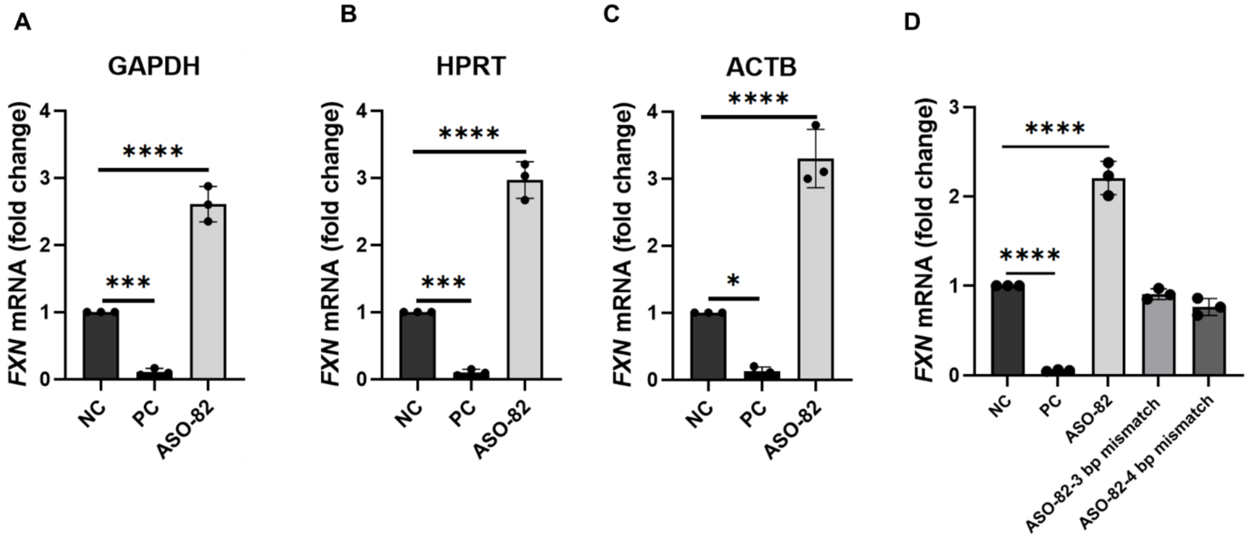

Figure S2: Specificity of ASO-82 effect on *FXN* mRNA expression. Comparative analysis of the effect of ASO-82 on *FXN* mRNA expression. The qRT-PCR data was normalized relative to the expression of **A**) Glyceraldehyde-3-phosphate dehydrogenase (*GAPDH*); **B**) Hypoxanthine-guanine phosphoribosyltransferase (*HPRT*) and **C**) Actin (*ACTB*) mRNAs. GAA/PM fibroblasts were transfected with 30 nM ASO followed by RNA isolation 72 h post-transfection. **D**) Effect of mismatches on ASO-82 efficacy. The GAA/PM fibroblasts were transfected with 30 nM scramble (NC), gapmer (PC), ASO-82 and ASO-82 with 3 bp or 4 bp mismatches. In all experiments, data for *FXN* mRNA were collected from three independent experiments (n = 3). Error bars indicate SD. Statistical significance was determined using one-way ANOVA with post-hoc Dunnett tests, with significant *p* values indicated as: **p*<0.5, ****p* <0.001, *****p*<0.0001.

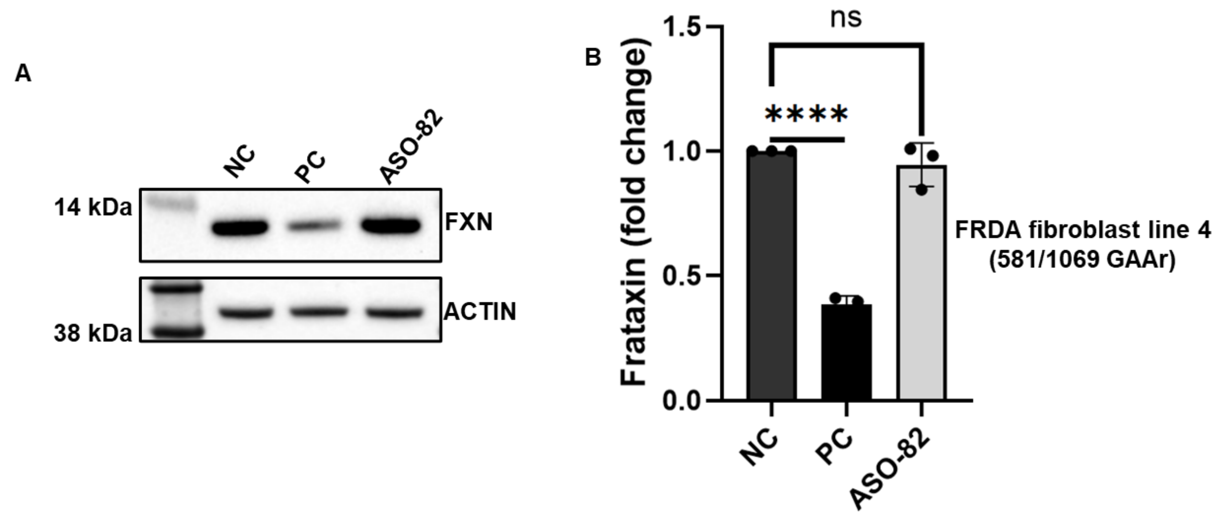

Figure S3: No effect of ASO-82 on FXN protein in FRDA fibroblasts carrying homozygous GAAr expansions. A-B) Frataxin level analyzed by western blot of homozygous GAAr expansion fibroblasts treated with 30 nM of ASO-82, NC, PC ASOs. Actin was used as a loading control and for normalization. Data in bar graphs represent mean values ± SD (n = 3). Statistical significance was determined using one-way ANOVA with a post-hoc Dunnett test, with significant *p* values indicated as: ****p<0.0001.

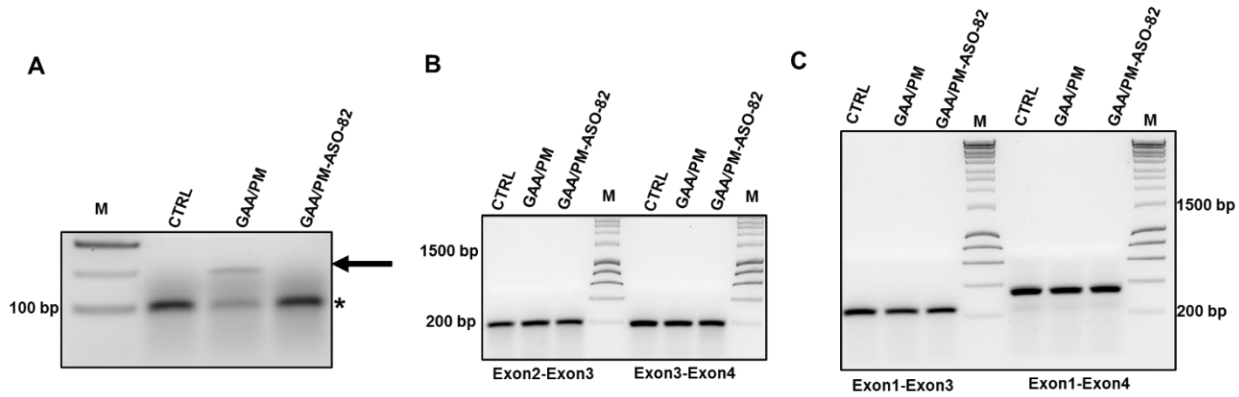

Figure S4: ASO-82 reduces expression of the aberrant transcript in *FXN* c.165 +5G>C cells without affecting splicing of the remaining introns in the *FXN* transcript. A) Transfection of GAA/PM cells with ASO-82 reduces levels of the aberrant transcript (indicated by the arrow) below the level of detection. The asterisk indicates RT-PCR product amplified from *FXN* early terminated transcript (*FXN-ett* RNA).[1] **B**) Amplification of the correctly spliced *FXN* transcript (exon 2 – exon 3 junction and exon 3 – exon 4 junction) in unaffected control (CTRL), GAA/PM and GAA/PM cells treated with ASO-82. **C**) Amplification of the *FXN* transcript confirming canonical inclusion of exon 2 (exon 1 – exon 3) as well as exons 2 and 3 (exon 1 – exon 4) in control (CTRL), GAA/PM and GAA/PM cells treated with ASO-82.

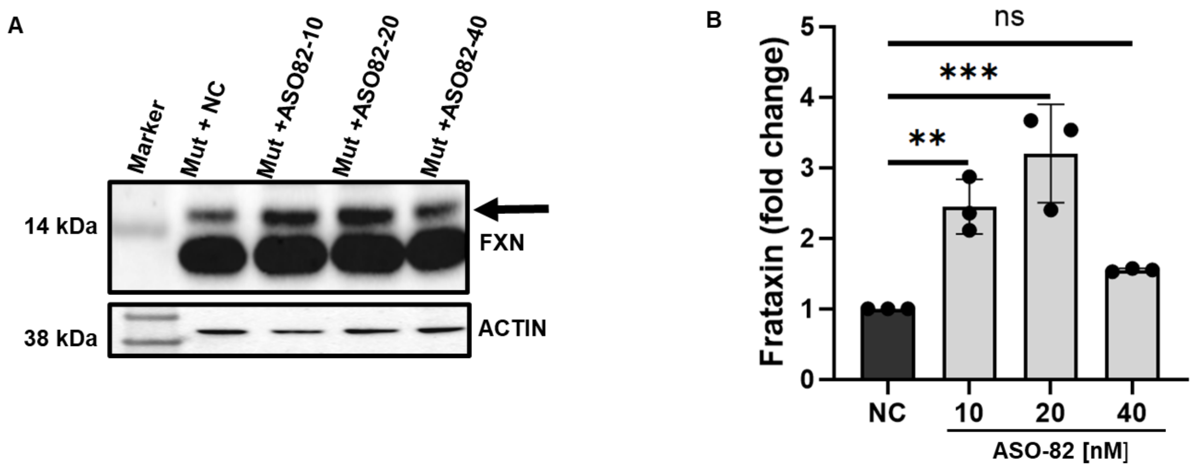

Figure S5: ASO-82 increases mini*FXN* expression in HEK293T cells in a dose-dependent manner. A-B) Western blot analysis shows exogenous FXN protein levels in the presence of increasing concentrations of ASOs up to 40 nM compared to the NC ASO. Toxicity was observed at the 40 nM concentration as reflected by the decrease in frataxin expression. Actin was used as a loading control and for normalization. Data in bar graphs represent mean values ± SD (n = 3). Statistical significance was determined using one-way ANOVA with a post-hoc Dunnett test, with significant *p* values indicated as: ***p*<0.01, ****p*<0.0001.

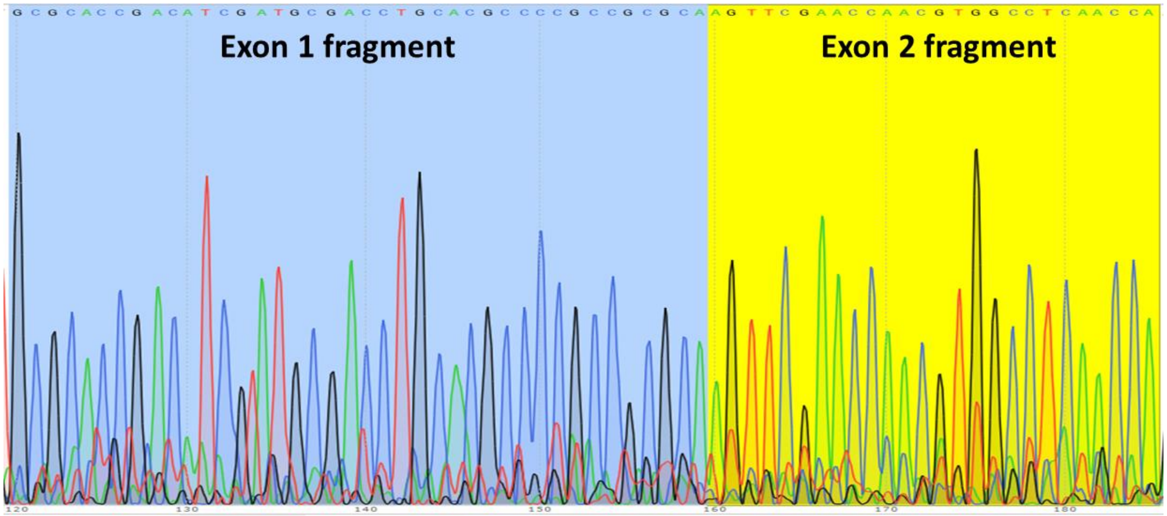

Figure S6: Splicing product 1 of WT and mutant *FXN* minigenes. Sanger sequencing of the RT-PCR product 1 (Fig. 6) representing canonical splicing of mini*FXN* (WT sequence).

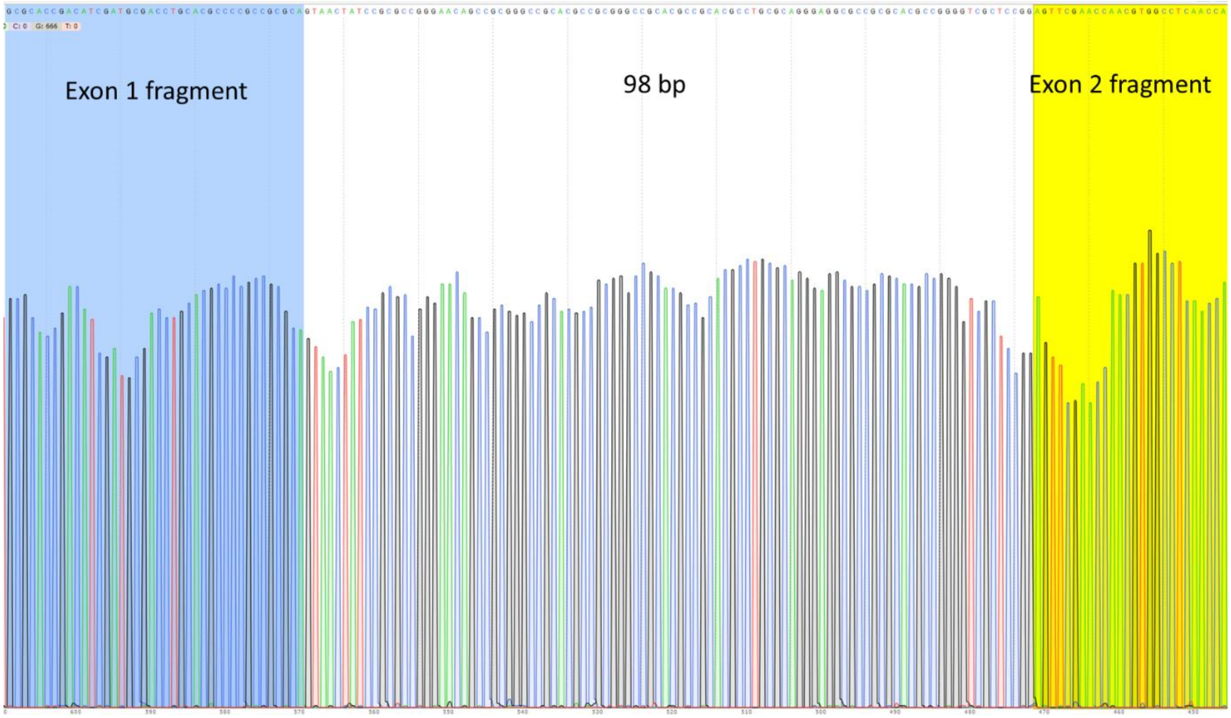

Figure S7: Splicing product 2 of WT and mutant *FXN* minigenes. Sanger sequencing of RT-PCR product 2 (Fig. 6) representing an aberrantly spliced transcript generated from c.165+5G>C mutated mini*FXN*. It includes partial retention of intron 1 (98 bp).

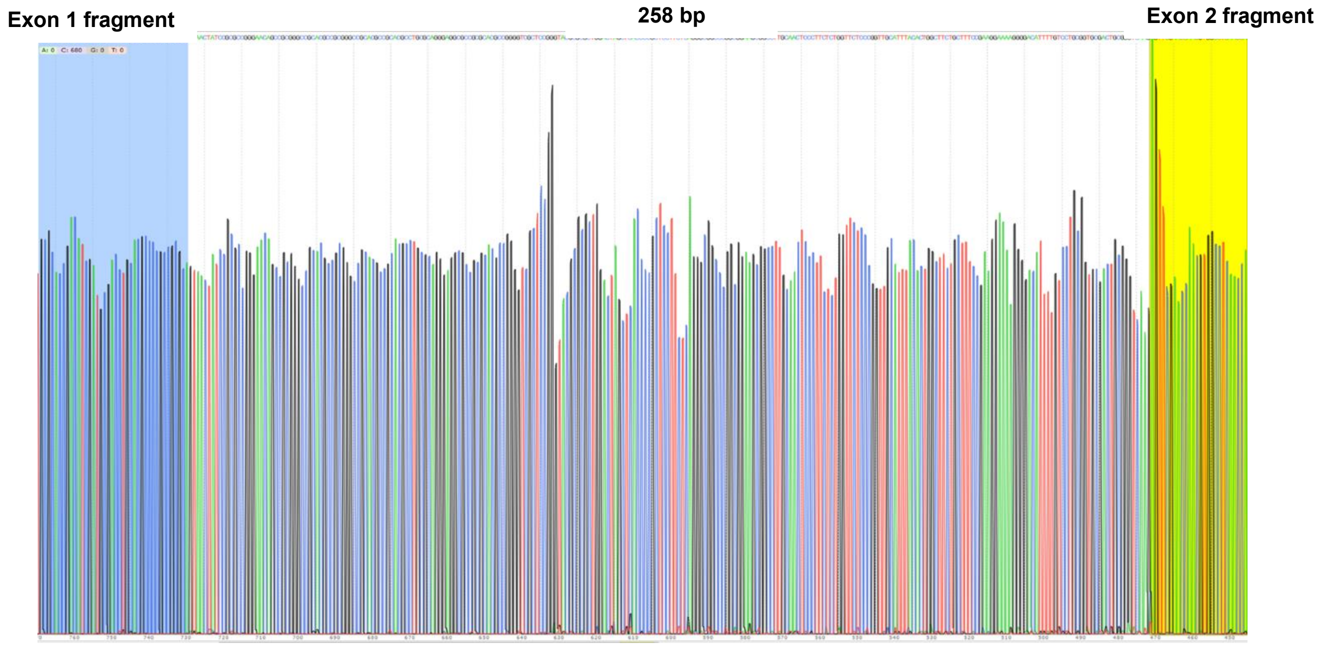

Figure S8: **Splicing product 3 of WT and mutant *FXN* minigenes.** Sanger sequencing of RT-PCR product 3 (Fig. 6) representing a second aberrantly spliced transcript generated from c.165+5G>C mutated mini*FXN*. It includes partial retention of intron 1 (258 bp).

Supplementary tables

Table S1. ASOs used in this study.

**Name**	**Sequences (5'-3')**
**First panel of ASOs**	
ASO-81	GCGGATACTTACTGCGCG
ASO-82	CGGCCCGCGGCTGTTCCC
ASO-83	CGGCTGTTCCCGGCGCGG
ASO-84	CGGCGTGCGGCCCGCGGC
ASO-85	TCCCGGCGCGGATACTTA
**Second panel of ASOs**	
ASO-1	TGTTCCCGGCGCGGATAG
ASO-2	CTGTTCCCGGCGCGGATA
ASO-3	GCTGTTCCCGGCGCGGAT
ASO-4	GGCTGTTCCCGGCGCGGA
ASO-83*	CGGCTGTTCCCGGCGCGG
ASO-6	GCGGCTGTTCCCGGCGCG
ASO-7	CGCGGCTGTTCCCGGCGC
ASO-8	CCGCGGCTGTTCCCGGCG
ASO-9	CCCGCGGCTGTTCCCGGC
ASO-10	GCCCGCGGCTGTTCCCGG
ASO-11	GGCCCGCGGCTGTTCCCG
ASO-82*	CGGCCCGCGGCTGTTCCC
ASO-13	GCGGCCCGCGGCTGTTCC
ASO-14	TGCGGCCCGCGGCTGTTC
**Controls**	
ASO-CTRL (NC)	GCTATACCAGCGTCGTCAT
ASO-GAPMER (PC)	GGCATAAGACATTAT
ASO-82-3	CGGACCGTGGCGGTTCCC[#]
ASO-82-4	CGACCTGCGGCTCTTACCC[#]

* repeated synthesis of the indicated ASOs
[#] nucleotides mutated in comparison to ASO-82 are underlined

Table S2. Primers used in this study.

Method	Name	Orientation	Sequence (5' – 3')
**PCR**			
	GAAr expansion (Fig. 1A)	Forward	GGAGGGAACCGTCTGGGCAAAGG
		Reverse	CAATCCAGGACAGTCAGGGCTTT
	PM region amplification (Fig. 1B)	Forward	AGCAGCATGTGGACTCTCG
		Reverse	TCCCCTTTTCCTTCGGAAAGC
	Primer short transcript (Fig. 2C, E)	Forward	ACCGACATCGATGCGACC
		Reverse	TCACACCAGGTCCGCAAAAT
	mini*FXN* mRNA (Fig. 6A, B)	Forward	CTTACGACGTGCCCGACTAC
		Reverse	TCCATCACCAGTATCCGAGC
	Exon 2 - Exon 3 of FXN (Fig. S4B)	Forward	GAACCAACGTGGCCTCAACC
		Reverse	TGTATGGCTTGTCTGCAAGGT
	Exon 3 - Exon 4 of FXN (Fig. S4B)	Forward	CAGAGGAAACGCTGGACTCT
		Reverse	AGCCAGATTTGCTTGTTTGG
	Exon 1 - Exon 3 of FXN (Fig. S4C)	Forward	ACCGACATCGATGCGACC
		Reverse	TGTATGGCTTGTCTGCAAGGT
	Exon 1 - Exon 4 of FXN (Fig. S4C)	Forward	ACCGACATCGATGCGACC
		Reverse	AGCCAGATTTGCTTGTTTGG
**qRT-PCR**			
	FXN mRNA	Forward	CAGAGGAAACGCTGGACTCT
		Reverse	AGCCAGATTTGCTTGTTTGG
	GAPDH mRNA	Forward	GAAGGTGAAGGTCGGAGTC
		Reverse	GAAGATGGTGATGGGATTTC
	HPRT mRNA	Forward	TGACACTGGCAAAACAATGCA
		Reverse	GGTCCTTTTCACCAGCAAGCT
	ACTB mRNA	Forward	CTGGAACGGTGAAGGTGACA
		Reverse	AAGGGACTTCCTGTAACAATGCA

References:

1. Li, Y., Li, J., Wang, J., Zhang, S., Giles, K., Prakash, T.P., Rigo, F., Napierala, J.S., Napierala, M. “Premature transcription termination at the expanded GAA repeats and aberrant alternative polyadenylation contributes to the Frataxin transcriptional deficit in Friedreich's ataxia” Hum Mol Genet. 2022 Jun 16; PMID: 35708503.
